# Averting Disease

**Published:** 1860-04

**Authors:** 


### AVERTING DISEASE.

THE very instant the scientific engineer observes any thing is wrong on ship, or train, or engine, he cuts off the supply of steam; so the very moment there is any sensation about the body sufficiently decided to attract the attention unpleasantly, that very moment should all supply of food be cut off; not an atom should be swallowed, at least until there has been time to ascertain the exact nature of the trouble.

If cutting off the supply of steam is not adequate to the rectification of the mischief, the next step taken is to work off the steam already generated; so, if abstinence from food is not sufficient to remove a given symptom or ailment, means should be taken to diminish the amount of that which the food previously eaten has made, that is, blood, including waste.

Pain is a blessing; it is the great life-preserver; it is the sleepless, faithful sentinel which gives prompt warning that harm is being done. All pain is experienced through the nerves; they telegraph it to the brain, and there the mind takes note of it. Pain is the result of pressure on or against a nerve; that pressure is made by a blood-vessel, for there is no nerve without a blood-vessel in close proximity. A blood-vessel is distensible, like an India-rubber life-preserver—both may be full and yet may be fuller. In health each blood-vessel is moderately full; but the very moment disease, or harm, or violence, by blow or cut or otherwise, comes to any part of the body, nature becomes alarmed as it were, and sends more blood there to repair the injury—much more than is usually required; that additional quantity distends the blood-vessels, and gives disquiet or actual pain. In these cases this increased quantity of blood is called "inflammation;" and if there is not this increased flow to the injured part, there is no healing, and that part dies, unless some stimulating application is made.

But pain comes in another way. If a man eats too much, or is constipated, or by some other means makes his blood impure, it becomes thickened thereby, and does not flow through its channels as freely as it should; hence it accumulates, dams up, congests, distending the veins, which in their turn make pressure on some adjoining nerve, and give dull pain. This congestion in the arteries gives a sharp or pricking pain.

Pain, then, is the result of more blood being determined to the part where that pain is, than naturally belongs to it. The evident alternative is to diminish the quantity of blood, either at the point of ailment or in the body in general. Thus it is that a mustard-plaster applied near a painful spot, by withdrawing the blood to itself, gives instantaneous relief. Opening a vein will do the same thing; and so, but not as expeditiously, will any purgative medicine, because that by all these things, by diminishing the amount of fluid as to the whole body, each particular part is proportionably relieved. On the same principle is it that a "good sweat" is "good" for any pain, and affords more or less relief. Friction does the same, even if it is performed with so soft a thing as the human hand, for any rubbing reddens, that is, attracts blood to the part rubbed, and thus diminishes the amount of pain at the spot where there is too much blood.

But the safer, more certain and durable method of relieving pain is to do it in a natural way, without the violence of the lancet, or the blister-plaster, or the purgative; and that is, by diminishing the amount of blood in the body, by cutting off the supply of its manufacture. The blood is made out of the food we eat, and it is just as easy to make a world out of nothing as to make more blood in the body without eating more. Ceasing to eat would be of itself a negative remedy—its only effect would be not to increase the pain; but nature's forces are always in operation; she is constantly engaged in unloading the body of its surplus fluids—unloading it in a million places at the same time, and in a million ways; every pore of the skin, at every instant of our existence, is discharging its portion of the substance of the body in the shape of insensible perspiration; and besides this, every breath we breathe, every emotion of the mind, every movement of a muscle, down to the crook of a finger or wink of an eye, is at the expense of atoms of the body; it contains less, weighs less, than at the instant before. Thus it is that if, in any pain, we instantly stop eating, and thus stop adding to the quantity of blood already in the body, nature will perform the other part, and diminish the supply every instant. So that the great remedy for pain is to lie still, wait and do nothing—the very course which blind instinct, by the wise and loving Father of us all, points out to wounded bird and beast and creeping thing, and they get well amain.

The great thing, then, to do in order to ward off serious disease, (and sickness never comes without a friendly premonition in the distance, only that in our stupidity or heedlessness we often fail to make a note of it,) is simply to observe three things.

1. The instant we become conscious of any unpleasant sensation in the body, cease eating absolutely.
2. Keep warm.
3. Be still.

These are applicable and safe in all cases; sometimes a more speedy result is attained if, instead of being quiet, the patient would, by moderate, steady exercise, keep up a gentle perspiration for several hours. And an observant person will seldom fail to discover that he who relies on a judicious abstinence and moderate exercise for the removal of his "symptoms," will find in due time, in multitudes of cases, that the remedy will become more and more efficient, with increasing intervals for need of its application, until at length a man is not sick at all, and life goes out like the snuff of a candle or as gently as the dying embers on the hearth.

